# Cardiac soluble guanylate cyclase protects against the cardiac dysfunction induced by chronic doxorubicin treatment in mice

**DOI:** 10.1186/2050-6511-16-S1-A96

**Published:** 2015-09-02

**Authors:** Sara Vandenwijngaert, Melissa Swinnen, Hilde Gillijns, Ellen Caluwé, Robert E Tainsh, Daniel I Nathan, Kaitlin Allen, Jozef Bartunek, Peter Brouckaert, Marielle Scherrer-Crosbie, Kenneth D Bloch, Stefan P Janssens, Emmanuel S Buys

**Affiliations:** 1 Department of Anesthesia, Critical Care, and Pain Medicine, Massachusetts General Hospital and Harvard Medical School, Boston, MA 02114, USA; 2 Department of Cardiovascular Sciences, KU Leuven, Leuven, 3000, Belgium; 3 Cardiovascular Center, OLV Hospital, Aalst, 9300, Belgium; 4 Department of Biomedical Molecular Biology, Ghent University and Flanders Institute for Biotechnology, Ghent, 9000, Belgium

## Background

The use of doxorubicin (DOX), a potent chemotherapeutic agent, is limited by cardiotoxicity, leading to congestive heart failure in up to 5% of DOX-treated patients. Dysfunctional cyclic guanosine 3’, 5’-monophosphate (cGMP) signaling has been implicated in various cardiovascular diseases, including cardiotoxicity associated with DOX administration. We tested the hypothesis that cGMP generated by soluble guanylate cyclase (sGC), the target for clinically available pharmacological agents that enhance cGMP levels (e.g. riociguat), protects against DOX-induced cardiomyopathy.

## Methods and results

Nitric oxide (NO)-stimulated sGC enzyme activity was lower in myocardial tissue extracts from wild-type (WT) mice exposed to DOX (20 mg/kg, IP, 24h), than from vehicle-treated WT mice (20.4±2.1 vs. 25.7±1.4 pmol cGMP/mg protein/min, respectively, n=10 for both, *P*<0.05). To investigate whether decreased cardiac cGMP synthesis by sGC contributes to DOX-induced cardiotoxicity, we studied mice with a cardiomyocyte-specific deficiency in the α1-subunit of sGC (sGCα1^-/-^), obtained using a Cre-lox conditional knockout strategy. At baseline, left ventricular (LV) dimensions and function, assessed via transthoracic echocardiography (TTE), were similar in sGCα1^-/-^ and WT mice. After 12 weeks DOX (2 mg/kg/week, IP), TTE and invasive hemodynamic measurements revealed greater LV dysfunction and dilatation in sGCα1^-/-^ than in WT mice (Table 1).

**Table 1:** DOX-induced LV dysfunction and remodeling after 12 weeks chronic treatment is greater in sGCα1^-/-^ than WT mice:

*Echocardiography*	**WT + saline** (n=7)	**sGCα1-/- + saline** (n=6)	**WT + DOX** (n=12)	**sGCα1-/- + DOX** (n=11)
LVID_S_ (mm)	1.3±0.1	1.3±0.0	1.7±0.1†	2.0±0.1†*
LVID_D_ (mm)	3.0±0.0	3.0±0.0	3.2±0.1†	3.3±0.0†
FS (%)	57±1	57±1	48±2†	40±1†*
HR (bpm)	531±13	505±18	428±17†	426±18†

***Hemodynamic measurements***	**WT + saline** (n=6)	**sGCα1-/- + saline** (n=5)	**WT + DOX** (n=12)	**sGCα1-/- + DOX** (n=9)

ESV (μl)	19±1	19±1	26±3†	37±3†*
EDV (μl)	43±2	41±2	51±2†	58±2†*
EF (%)	60±2	58±3	55±4	42±3†*
dP/dt_max_ (mmHg/s)	13,499±1,433	11,995±601	13,503±678	10,386±875*
dP/dt_min_ (mmHg/s)	-12,053±1,809	-11,420±950	-13,531±846	-10,943±940¶
Tau (ms)	5.1±0.1	4.9±0.2	5.6±0.2†	6.3±0.3†¶
PRSW	83±9	84±6	70±9	49±7†¶
Ees (mmHg/μl)	6.3±1.2	7.3±1.4	4.0±0.8†	2.3±0.3†¶
EDPVR (mmHg/μl)	0.21±0.03	0.18±0.02	0.16±0.02	0.19±0.02
HR (bpm)	576±8	614±12	528±9†	530±14†*

LVID_S_ indicates left ventricular internal diameter at end-systole; LVID_D_, left ventricular internal diameter at end-diastole; FS, fractional shortening; HR, heart rate; ESV, end-systolic volume; EDV, end-diastolic volume; EF, ejection fraction; dP/dt_max_ and dP/dt_min_, maximum and minimum of the first derivative of LV pressure over time; Tau, time constant for isovolumic relaxation; PRSW, pre-load recruitable stroke work; Ees, end-systolic elastance; and EDPVR, end-diastolic pressure volume relationship. Values are presented as mean±SEM.

^†^*P*<0.05 vs. saline,

* *P*<0.05 vs. WT + DOX, and

^¶^*P*≤0.10 vs. WT + DOX.

In a second mouse model, myocardial sGC activity was reduced by cardiomyocyte-specific expression of a dominant-negative sGCα1 mutant (DNsGCα1^tg/+^) in an inducible manner (Tet-Off). Withdrawing tetracycline from the diet resulted in a ~50% reduction of cardiac sGC activity (18.6±2.6 vs. 32.0±5.4 pmol cGMP/mg protein/min in 8 DNsGCα1^tg/+^and 8 WT mice, respectively*, P*<0.05). At baseline, LV dimensions and function were similar in DNsGCα1^tg/+^and WT mice. Chronic DOX treatment resulted in greater LV systolic dysfunction and dilatation in DNsGCα1^tg/+^ than in WT mice after 8 and 12 weeks (TTE, data not shown). Importantly, LV dysfunction observed in DNsGCα1^tg/+^exposed to DOX for 12 weeks could be attenuated by re-adding tetracycline to the diet [thereby suppressing expression of the dominant negative sGCα1 mutant and de-repressing endogenous sGC activity] after 8 weeks of DOX administration: fractional shortening improved significantly in DNsGCα1^tg/+^ mice by 16 weeks (28±1% at 8 weeks vs. 35±1% at 16 weeks, n=17 and 20, respectively, *P* <0.05).

## Conclusions

Reduced myocardial sGC activity results in increased LV dysfunction and dilatation in DOX-treated mice. Pharmacological stimulation of sGC may represent a promising therapeutic approach to tackle DOX-associated cardiotoxicity.

